# NMR-Based Metabolomic Approach to Study Growth of *Phaseolus vulgaris* L. Seedlings Through Leaf Application of Nanofertilizers and Biofertilizers

**DOI:** 10.3390/ijms26104844

**Published:** 2025-05-19

**Authors:** Elsy Rubisela López-Vargas, Diego Hidalgo-Martínez, Elvia Becerra-Martínez, L. Gerardo Zepeda-Vallejo, Claudia J. Hernández-Guerrero, Alma Delia Hernández-Fuentes, Gregorio Cadenas-Pliego, Marissa Pérez-Álvarez

**Affiliations:** 1 Institute of Agricultural Sciences, Autonomous University of the State of Hidalgo, Tulancingo 43600, Mexico; elsy_lopez@uaeh.edu.mx; 2 Department of Biology, Healthcare and the Environment, Faculty of Pharmacy and Food Sciences, University of Barcelona, 08028 Barcelona, Spain; dhidalgo@ub.edu; 3 Centro de Nanociencias y Micro y Nanotecnologías, Instituto Politécnico Nacional, Av. Luis Enrique Erro S/N, Unidad Profesional Adolfo López Mateos, Zacatenco, Delegación Gustavo A. Madero, México City 07738, Mexico; 4 Department of Organic Chemistry, National School of Biological Sciences, National Polytechnic Institute, Prolongación de Carpio y Plan de Ayala S/N, Col. Santo Tomás, Delegación Miguel Hidalgo, México City 11340, Mexico; lzepeda@ipn.mx; 5 Instituto Politécnico Nacional, Centro Interdisciplinario de Ciencias Marinas, Av. Instituto Politécnico Nacional S/N, Col. Playa Palo de Santa Rita, La Paz 23096, Mexico; cguerrer@ipn.mx; 6 Research Center for Applied Chemistry, Macromolecular Chemistry and Nanomaterials, Saltillo 25294, Mexico; gregorio.cadenas@ciqa.edu.mx (G.C.-P.); marissa.perez@ciqa.edu.mx (M.P.-Á.)

**Keywords:** nanofertilizers, biofertilizers, black beans, metabolomics, nuclear magnetic resonance

## Abstract

This study investigated the effects of two nanofertilizers (NFs): copper nanoparticles (NPs) synthesised using cotton (CuC) and chitosan (CuCh) as well as two biofertilizers (BFs), nopal extract (NE) and commercial Biojal^®^ worm humus (WH), on the growth of black bean seedlings. The treatments consisted of applying 50 mg L^−1^ of CuC, 50 mg L^−1^ of CuCh, 50 mg L^−1^ of NE, 100 mg L^−1^ of WH, their respective combinations, and an absolute control that consisted of distilled water. The CuC, CuCh, WH, and WH + CuC leaf applications resulted in an increase in plant height by 34.4%, 19.5%, 25.7%, and 20.3%, respectively. Furthermore, the CuC and WH applications led to an increase in the number of leaves by 53.2% and 36.9%, respectively. However, the addition of NE + CuC resulted in a 37.4% decrease in dry weight. A total of 44 metabolites were identified, including 7 sugars, 17 amino acids, 12 organic acids, 4 nucleosides, 1 alcohol, and 3 miscellaneous metabolites. The NE + CuC and WH treatments resulted in a notably higher concentration of various metabolites, including amino acids, organic acids, and sugars. Conversely, the CuCh treatment led to an increased concentration of nucleosides, amino acids, trigonelline, and nicotinamide adenine dinucleotide (NAD^+^).

## 1. Introduction

The agricultural industry is currently facing a crisis due to climate change and the overutilisation of chemical fertilizers and pesticides [1]. Conventional fertilizers negatively affect soil health and the overall ecosystem [2]. However, nanotechnology has the potential to reduce the environmental impact of traditional farming practices [3]. Nanotechnology has demonstrated its capacity to revolutionise agricultural systems via advancements in crop production through the use of nanofertilizers (NFs) and nanopesticides [4]. Nanofertilizers are composed of nanoparticles (NPs) containing macro- or micronutrients that are strategically applied to crops to improve both the plants’ growth and quality [5] in a controlled manner. Furthermore, NFs enhance nutrient absorption efficiency, boost photosynthesis, reduce soil toxicity, lower the required application frequency, and, most importantly, mitigate environmental pollution [6]. However, it is important to note that producing NPs involves using chemicals that consume energy and produce environmental pollution [3].

Various biological synthetic technologies have recently emerged for manufacturing NPs; such technologies are renowned for being environmentally friendly, efficient, and non-toxic. Nanoparticles obtained via biological or green synthesis routes generate less waste, rendering them effective and ecological methods for use with agricultural crops [7,8]. One of the metals that can be synthesised and used as a NFs is copper (Cu) [9].

Copper is an essential micronutrient vital for plant growth and metabolism [10]. Its presence improves chlorophyll formation, and Cu is involved in cell-wall development; the element also exhibits antimicrobial properties and provides resistance to stress [11]. Additionally, Cu participates in enzymatic reactions and serves as a cofactor for several proteins [12]. Some authors suggests that the use of copper oxide (CuO) NPs can increase plant growth due to enhanced bioavailability of Cu^2+^; these NPs are accordingly efficient NFs [3]. Other authors applied CuO NPs (80 mg L^−1^) to dragonhead plant leaves and observed an increase in biomass, elevated accumulation of Cu in shoots, and higher yields of bioactive compounds [13]. Similarly, foliar application of CuO NPs (8 mg L^−1^) to mustard plants was evaluated. The results indicated significant increases in growth and biomass, as well as an increased content of chlorophylls and proline and heightened antioxidant activity [11].

On the other hand, biofertilizers (BFs) are another alternative to improve plant nutrition, promote soil health, and optimise crop yields for sustainable agricultural production [14,15]. However, the effectiveness of biofertilization in agriculture is influenced by various factors. Some authors have noted that the combination of NFs with BFs increases nutrient-utilisation efficiency and improves tolerance to abiotic stress [16].

The common bean (*Phaseolus vulgaris* L.) is one of the most important legumes in the world [17]. In Mexico, it is one of the basic foods in the local food basket; it serves as a staple in the diets of lower-income individuals due to the high nutritional value it provides at a low cost. The common bean furthermore plays a vital role in children’s diets as it promotes brain development owing to its rich content of iron, protein, calcium, and fibre [18]. In addition, it is an important source of protein (14 to 33%), starch, B complex vitamins, minerals (calcium, copper, potassium, magnesium, phosphorus and zinc), and dietary fibre [18,19].

The use of NPs in plants confers promise for improving crop qualities and yields, given that plants activate defence mechanisms to adapt to adverse environmental conditions. Such a situation can lead to the accumulation or depletion of various metabolites [20]. Changes in the metabolic profiles of plants can arise from genetic modifications, external stimuli, or exposure to stressors or elicitors [21,22]. These changes can be effectively studied using a metabolomic approach, which involves analysing the metabolites that serve as biomarkers during stressful situations [23]. Metabolomic analysis can involve several different techniques. Nuclear magnetic resonance (NMR) spectroscopy is a rapid, reproducible, and high-throughput technique [24,25]. ^1^H NMR, combined with multivariate data analysis, is one of the most-used methodologies for metabolomics studies [26].

We assumed that the foliar application of NFs or BFs could enhance plant nutrition while minimising the negative consequences associated with the excessive use of conventional chemical fertilizers. Furthermore, the combination of NFs and BFs has the potential to amplify their respective effects on plant growth and development [24]. Therefore, the objective of this work was to assess the impact of foliar application of Cu NPs synthesised using green methods; these NPs functioned as both NFs and BFs. We furthermore investigated their synergistic effects on the agronomic and metabolomic responses of black bean seedling leaves.

## 2. Results

### 2.1. Bean Crop Growth

The application of nanofertilizers (NFs) and biofertilizers (BFs) to the leaves of black bean seedlings resulted in significant modifications to the plant height (PH), number of leaves per plant (NL), and dry weight (DW). The copper-cotton (CuC) and copper-chitosan (CuCh) NPs exhibited notable increases in PH (i.e., 34.4% and 19.5%, respectively, compared with the absolute control (C)). Among the BFs, only Biojal^®^ worm humus (WH) showed a substantial increase of 25.7%. The combination of NFs and BFs did not significantly affect PH, with the exception of the WH + CuC treatment, which resulted in a 20.3% increase in PH compared to the absolute control (C) (Figure S1A).

Both stem diameter (SD) and fresh weight (FW) were unaffected by any of the treatments (Figure S1B,C). However, the NL increased by 53.2% and 36.9% after the CuC and WH treatments, respectively (Figure S1D). The DW decreased by 37.4% relative to the absolute control (C) after the NE + CuC treatment (Figure S1E).

### 2.2. Identification of Metabolites

Figure S2 shows the ^1^H NMR spectrum (750 MHz) of the aqueous extract from untreated black beans leaves (C). We recorded multiple signals from 0.00 to 9.50 ppm belonging to 44 metabolites (i.e., 7 sugars, 17 amino acids, 12 organic acids, 4 nucleosides, 1 alcohol, and 3 miscellaneous metabolites). These findings are consistent with those reported by others authors [17], who studied the metabolic profile of seeds and leaves from five bean cultivars. When comparing the spectra, we obtained with those reported certain variations can be observed across different regions (Figure S2A–D). For instance, the signal intensity of 2-hydroxy-isobutyric acid was significantly higher over the range of 0.80–3.30 ppm (Figure S2B). Likewise, within 3.30–5.50 ppm range, more intense signals were observed for sugars (Figure S2C). Additionally, signals corresponding to amino acids and nucleosides appeared quite similar over the range 5.50–9.50 ppm; the exception was trigonelline, which exhibited a weaker signal (Figure S2D). Figure S7 illustrates the representative spectra of various treatments with NFs and BFs. It is noteworthy that the CuCh, WH, and NE + CuC treatments resulted in a higher signal intensity for various metabolites. This finding suggests that a greater concentration of metabolites persisted after those treatments compared with other treatments. For detailed information on the chemical shifts, multiplicity, and coupling constants of the metabolites identified in aqueous extracts of untreated black bean (*Phaseolus vulgaris* L.) leaves, the reader is referred to Table S2.

### 2.3. Multivariate Analysis

**Figure 1:**
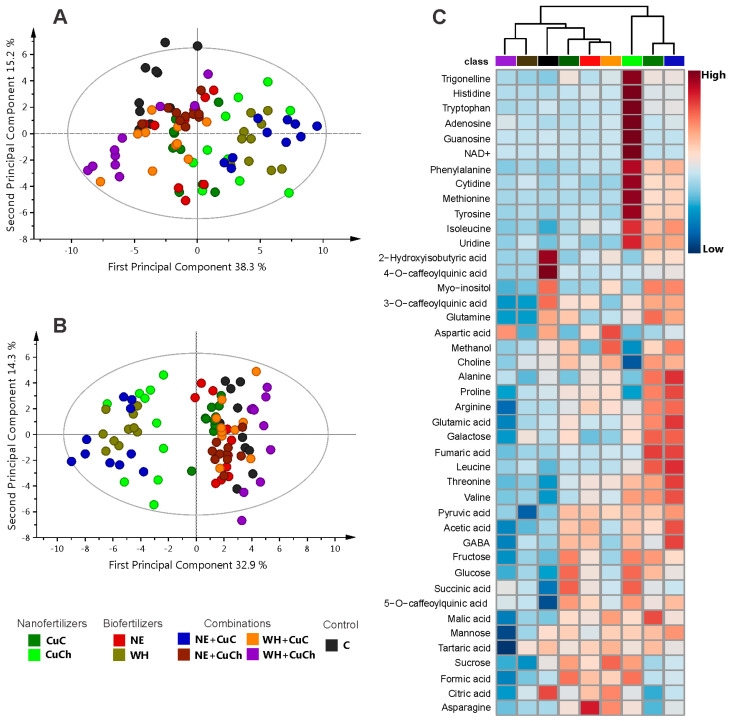
Scatter plot depicting PCA scores with outliers (**A**); scatter plots from OPLS-DA without outliers (**B**), both derived from NFs and BFs applied to black bean leaves (*Phaseolus vulgaris* L.). Heatmap showing the relative abundance of identified metabolites (**C**). Red denotes a high relative abundance; blue denotes a low relative abundance. C: absolute control; CuC: copper-cotton NPs; CuCh: copper-chitosan NPs; NE: nopal extract; WH: Biojal^®^ worm humus.

Figure 1 illustrates the results obtained from the principal component analysis (PCA) and orthogonal partial least squares discriminant analysis (OPLS-DA) models, along with the corresponding heat map derived from the ^1^H NMR spectra of black bean leaves treated with NFs and BFs. The PCA model was applied to assess the behaviour of the treatments. The result of the score scatterplot reveals no distinction among the treatments. The first principal component (PC1) and the second principal component (PC2) explained 38.3% and 15.2% of the variance, respectively (Figure 1A). On the other hand, the OPLS-DA graph reveals two-dimensional projections (PC1 = 32.9% and PC2 = 14.3%) indicating that the NE + CuC, WH, and CuCh treatments were distinct from the rest of the treatments (Figure 1B). The values of R^2^ and Q^2^, as well as the results of the permutation tests, can be found in Table S3. The heatmap (Figure 1C), by providing a visual representation of the metabolomic profile of each treatment, enables a comparison of the differences in relative concentration levels of the metabolites in the bean leaves. Seedlings treated with NE + CuC exhibit the highest concentration of metabolites, followed by the seedlings that received the WH and CuCh treatments. This finding is consistent with varied effects of the treatments observed in the OPLS-DA model. In particular, the NE + CuC and WH treatments resulted in a marked abundance of amino acids, organic acids, and sugars. The addition of CuCh also resulted in a high content of nucleosides, amino acids, trigonelline, and NAD^+^. On the other hand, when BFs (NE and WH) were used together with CuCh, the metabolite level fell significantly. This finding was also observed in the control group (C).

**Figure 2:**
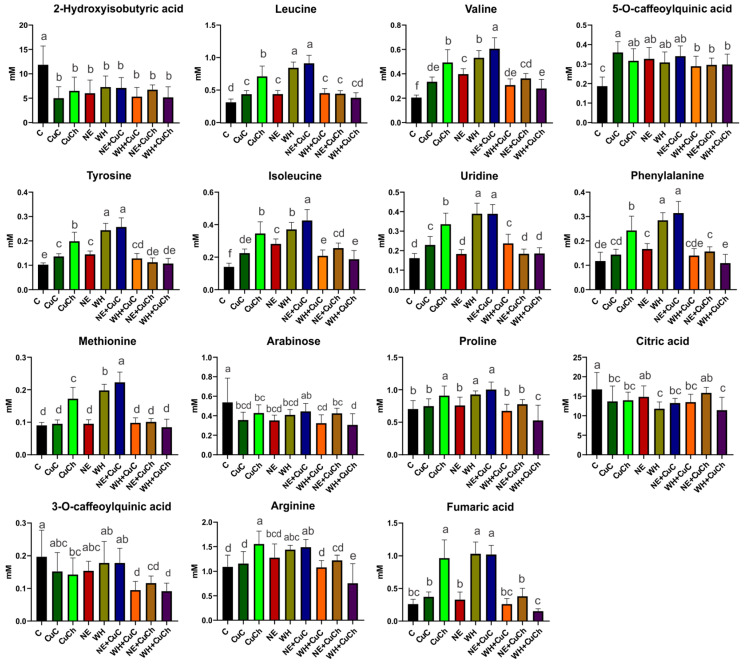
Relative concentrations of differential metabolites obtained the VIP scores of the OPLS-DA model for black bean leaves (*Phaseolus vulgaris* L.) treated with NFs and BFs and their respective combinations. Each data point represents the average of 10 replicates ± standard errors. Letters indicate significant differences between the treatments according to Duncan’s test (*p* ≤ 0.05). C: absolute control; CuC: copper-cotton NPs; CuCh: copper-chitosan NPs; NE: nopal extract; WH: Biojal^®^ worm humus.

The discriminant metabolites were selected based on the projection of important variables (VIP > 1) obtained from the OPLS-DA model. The foliar application of NFs and BFs, as well as their combination, resulted in changes in the concentration of certain metabolites present in the bean leaves. Specifically, the levels of eight amino acids, five organic acids, sugars (arabinose), and nucleosides (uridine) were affected. The treatments that resulted in the most significant metabolite increment included NE + CuC, WH, and CuCh. For instance, leucine increased by 192.68%, 170.62%, and 128.36%, respectively, after the NE + CuC, WH, and CuCh treatments. Valine increased by 195.13%, 159.21%, and 140.40%, respectively. Tyrosine increased by 150.98%, 138.18%, and 93.85%, respectively; isoleucine increased by 203.56%, 164.36%, and 146.33%, respectively. Phenylalanine increased by 167.01%, 141.92%, and 106.38%, respectively. Methionine increased by 146.43%, 118.68%, and 90.60%, respectively. Proline increased by 42.63%, 31.90%, and 29.24%, respectively. Arginine increased by 36.48%, 31.90%, and 42.44%, respectively. Fumaric acid increased by 290.94%, 295.16%, and 270.85%, respectively; uridine increased by 140.74%, 141.18%, and 107.93% (Figure 2).

5-O-Caffeoylquinic acid levels were higher after the CuC treatment compared with the absolute control (C). On the other hand, the addition of WH + CuC, CuCh, NE + CuCh, and WH + CuCh decreased the concentration of 3-O-caffeoylquinic acid by 51.88%, 27.69%, 41.02%, and 53.54%, respectively. A decrease in 2-hydroxyisobutyric acid was observed in all treatments; the CuC treatment resulted in the greatest reduction (i.e., 57.73% compared with the absolute control (C)). Citric acid levels were also affected; they decreased by 16.65–31.80% with the treatments.

The exception was NE and NE + CuCh, which resulted in no significant differences compared with the absolute control (C). Similarly, arabinose decreased with the application of NFs and BFs; the exception was the NE + CuC treatment, which was similar to the absolute control (C). It is worth noting that the WH + CuCh treatment resulted in the lowest arabinose content (i.e., a decrease of 43.00% compared with the absolute control (C) (Figure 2).

**Figure 3:**
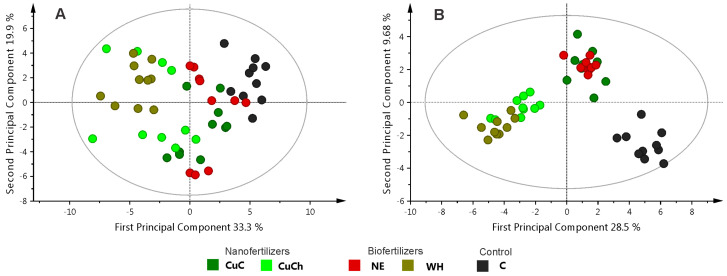
Principal component analysis score scatter plot with outliers (**A**) and OPLS-DA score scatter plots (**B**) for the leaves of black bean (*Phaseolus vulgaris* L.) treated with NFs and BFs. C: absolute control; CuC: copper-cotton NPs; CuCh: copperchitosan NPs; NE: nopal extract; WH: Biojal^®^ worm humus.

We used PCA and OPLS-DA to study the NFs and BFs treatments. Principal component analysis did not reveal clear differentiation among the treatments. PC1 and PC2 accounted for 33.3% and 19.9% of the variance, respectively (Figure 3A). Conversely, the graphical results of the OPLS-DA model (PC1 = 28.5% and PC2 = 9.68%) revealed that the treatments could be separated into two groups: Group 1 consisting of CuCh and WH, and group 2 consisting of C, CuC, and NE. However, it is noteworthy that C also exhibited discernible segregation from the CuC and NE treatments (Figure 3B).

**Figure 4:**
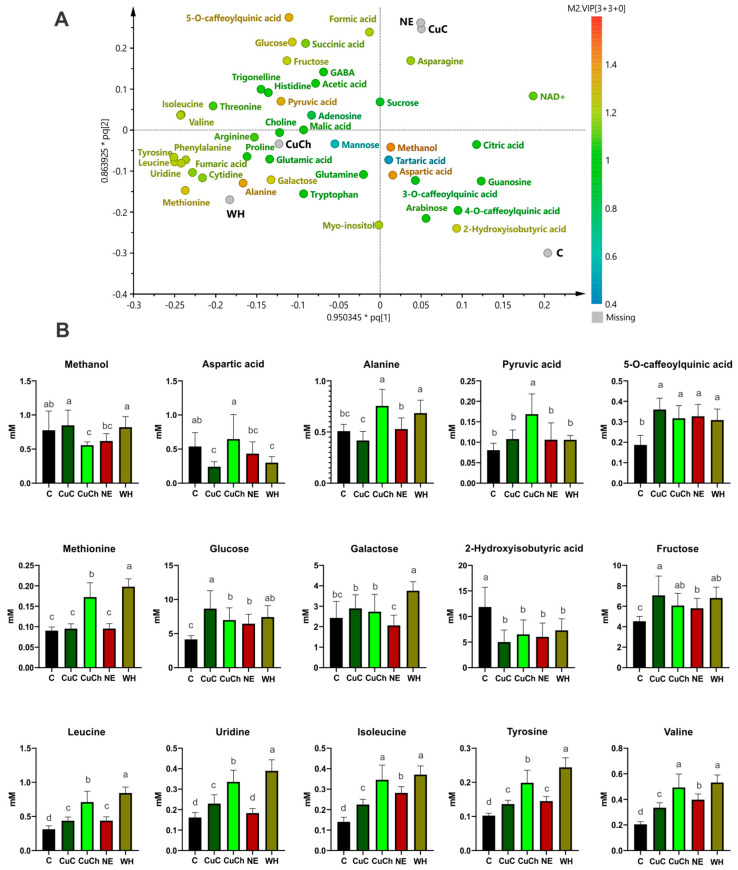
The corresponding loading of scatter plots generated from the ^1^H NMR spectra (750 MHz) of the metabolites found in black bean (*Phaseolus vulgaris* L.) leaves treated with NFs and BFs illustrated in (**A**). The relative concentrations of various metabolites as a function of VIP score from the OPLS-DA model (**B**). Each data point is the average of 10 replicates ± standard error. Letters indicate significant differences between the treatments according to Duncan’s test (*p* ≤ 0.05). C: absolute control; CuC: copper-cotton NPs; CuCh: copper-chitosan NPs; NE: nopal extract; WH: Biojal^®^ worm humus.

These groups self-segregated according to the concentration of metabolites resulting from the treatments; for instance, the CuC and NE treatments produced metabolites such as formic acid, asparagine, sucrose, and NAD^+^. The CuCh and WH treatments yielded the highest relative concentration of metabolites; the C samples exhibited higher concentrations of arabinose guanosine, methanol, citric acid, aspartic acid, tartaric acid, and caffeoylquinic acids (Figure 4A).

The differential metabolites obtained from the OPLS-DA model using a loading scatter plot are shown in Figure 4B. We found an increase in the content of 5-O-caffeoylquinic acid (92.30%), glucose (108.60%), fructose (55.69%), leucine (40.37%), uridine (41.98%), isoleucine (60.37%), tyrosine (32.91%), and valine (63.16%) after the CuC treatment. However, aspartic acid (55.39%) and 2-hydroxy-isobutyric acid (57.73%) decreased. On the other hand, we did not observe any significant differences in the content of methanol, alanine, pyruvic acid, methionine, or galactose compared with the C group.

We noted a higher concentration of alanine (48.53%), pyruvic acid (108.64%), tyrosine (93.85%), and valine (140.40%) after the CuCh treatment; there were also slight increases in 5-O-caffeolquinic acid (69.57%), methionine (90.60%), glucose (68.21%), fructose (33.84%), leucine (128.36%), uridine (107.93%), and isoleucine (146.33%). Conversely, methanol and 2-hydroxyisobutyric acid decreased by 28.12% and 45.16%, respectively, compared with the C group. Aspartic acid and galactose levels were similar to those observed in the C group.

The WH treatment resulted in the largest increase in metabolites of all the BFs. For instance, the contents of methionine (118.68%), galactose (54.69%), leucine (170.62%), uridine (141.18%), isoleucine (164.36%), tyrosine (138.18%), and valine (159.21%) all increased significantly. Additionally, alanine, 5-O-caffeoylquinic acid, glucose, and fructose exhibited increases of 34.38%, 64.97%, 78.77%, and 50.24%, respectively. Only aspartic acid and 2-hydroxyisobutyric acid decreased (by 44.05% and 38.55%, respectively). Methanol and pyruvic acid exhibited no differences compared with the C group.

We only observed slight increases in 5-O-caffeoylquinic acid (64.97%), glucose (55.24%), fructose (27.91%), leucine (40.88%), isoleucine (100.86%), tyrosine (41.50%), and valine (93.96%) for BFs with NE. The sole decrease was observed in 2-hydroxyisobutyric acid (49.27%); the levels of the remaining metabolites were similar to those of the C group (Figure 4B).

**Figure 5:**
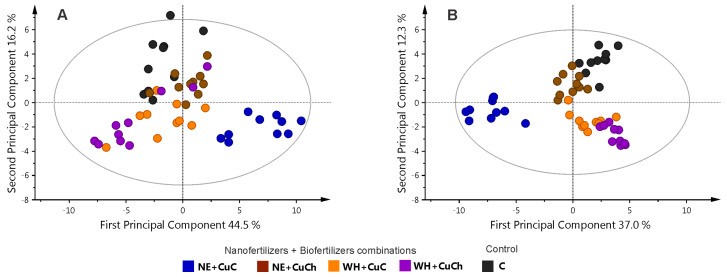
Principal component analysis score scatter plot with outliers (**A**) and OPLS-DA score scatter plots with outliers removed (**B**) for the leaves of black bean (*Phaseolus vulgaris* L.) treated with combinations of NFs and BFs. C: absolute control; CuC: copper-cotton NPs; CuCh: copper-chitosan NPs; NE: nopal extract; WH: Biojal^®^ worm humus.

Figure 5A shows a PCA model plot for the combination of NFs + BFs. The plot reveals a lack of discernible separation among the treatments; however, OPLS-DA revealed that the treatments could be divided into two distinct groups: the NE + CuC treatment was distinct from the rest of the treatments (Figure 5B).

**Figure 6:**
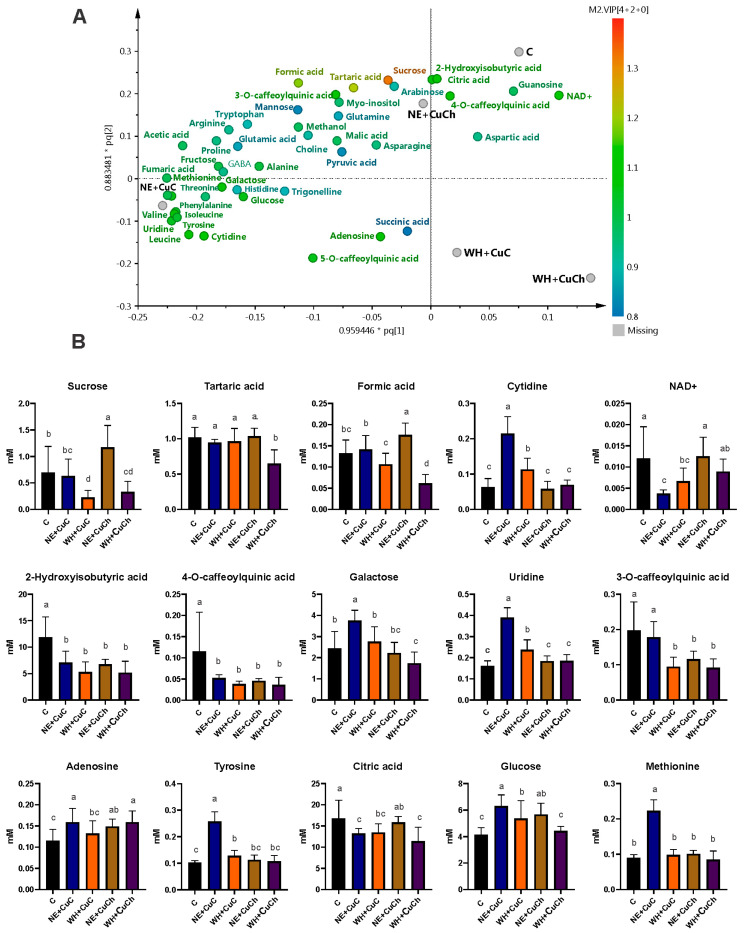
The corresponding loading of scatter plots generated from the ^1^H NMR spectra (750 MHz) of the metabolites found in black bean (*Phaseolus vulgaris* L.) leaves treated with NFs and BFs illustrated in (**A**). Relative concentrations of differential metabolites obtained as a function of VIP score from the OPLS-DA (**B**). Each data point is the average of 10 replicates ± standard error. Letters indicate significant differences between the treatments according to Duncan’s test (*p* ≤ 0.05). C: absolute control. C: absolute control; CuC: copper-cotton NPs; CuCh: copper-chitosan NPs; NE: nopal extract; WH: Biojal^®^ worm humus.

The NE + CuC treatment resulted in the highest concentration of metabolites; the NE + CuCh treatment followed next (Figure 6A). However, the combination of CuC and CuCh NFs with BFs and WH had a detrimental effect—both treatments resulted in a reduction in metabolites.

The combination of NE + CuC led to an increase in the content of sugars, including glucose (52.16%) and galactose (54.61%), as well as amino acids such as methionine (146.43%), and tyrosine (150.98%). The nucleosides cytidine (241.27%), uridine (140.74%), and adenosine (37.07%) increased as well. The content of organic acids such as 2-hydroxyisobutyric, 4-O-caffeoylquinic acid, citric acid, and NAD^+^ decreased with this combination, however. The remaining metabolites yielded levels similar to those of the C group.

The combination of NE + CuCh resulted in an increase in the contents of sucrose (67.29%), glucose (36.80%), formic acid (33.33%), and adenosine (28.45%). However, the combination led to a 43.09% decrease in the concentration of 2-hydroxyisobutyric acid. 3-O-caffeoylquinic acid also decreased by 41.02%, and 4-O-caffeoylquinic acid decreased by 60.34%. The remaining metabolites exhibited no significant differences compared with the C group. For the combination of WH + CuC, we noted an increase in the content of glucose (52.16%), tyrosine (25.00%), cytidine (79.37%), and uridine (46.87%); the treatment only increased the concentration of adenosine (37.07%). For both the NE + CuCh and WH + CuC combinations, the levels of the remaining metabolites did not significantly differ from those of the C group (Figure 6B).

## 3. Discussion

This study is the first to report a ^1^H NMR-based metabolomics profiling of black bean leaves, specifically investigating the effects of NFs and BFs foliar fertilization under field conditions. Prior to this research, a comprehensive metabolomic analysis of bean leaves was conducted, which established a protocol for replicating and validating the metabolic profile of this crop [17]. One advantage of foliar application of NFs is that NPs can pass through plant cells and penetrate the interior of the leaves given their nanometric size and the subsequent increase in their surface area [27]. As a result, nutrient penetration, absorption, and efficiency are enhanced [28]. On the other hand, conventional fertilizers only penetrate the leaf tissue of plants [29]. We have shown that the introduction of NPs increases plant leaf area. The increase expands the photosynthetic surface and increases the amount of chlorophyll and plant biomass, which can in turn trigger vigorous cumulative growth [30]. The observed increase in plant height and number of leaves can be attributed to the addition of CuC NPs.

Some authors have noted that the responses of plants exposed to BFs and nanomaterials in tandem differ from those observed when only BFs are applied [31]. This combination of elicitors can result in synergistic or antagonistic effects that can lead to improved plant resilience or a failure to withstand biotic or abiotic stresses [32]. Our findings demonstrate that both NFs and BFs influence the overall composition of metabolites in plants’ leaves. However, we noted an antagonistic effect when NFs and WH were combined—the combination led to a decrease in metabolite concentration in black bean leaves. That was unlike the combination of BFs of NE with CuC, which increased the concentration of metabolites. Also, the combination of NFs and BFs can prolong the lifespan and stability of nanoformulations against ultraviolet inactivation and heat [33]. Therefore, we assumed that the combination of BFs of NE with NFs of CuC would form the most stable nanoformulation and thereby elicit positive responses in the analysed crop.

Studying metabolites in plants is vital for gaining insights into their biology and understanding the effects of stress factors [34]. By monitoring changes in the concentrations of specific metabolites, it is possible to assess the impact of external stimuli and stressors [35]. We observed increased levels of organic acids, including pyruvic, acetic, fumaric, and succinic acids, when NE + CuC and CuCh were applied in tandem. These acids are of great importance in plants given that they participate in the tricarboxylic acid cycle, also known as the Krebs cycle, which generates energy for cellular processes [36]. Furthermore, an increase in organic acids triggers the increase or decrease in other metabolites, in this case an increase in pyruvic acid increases the concentration of tryptophan, which is a precursor of indole-3-acetic acid (IAA) through the indole-3-pyruvic acid pathway. IAA predominantly represents auxins, which is a major phytohormone that regulates cell division, expansion, and differentiation and is a regulator of cotyledon and leaf development [37], which could explain some changes in plant growth parameters such as height and leaf number.

Moreover, the addition of CuCh, CuC, NE + CuC, and WH positively affected the levels of sugars such as glucose, fructose, sucrose, galactose, and mannose. These sugars serve as energy sources and signal transduction factors in stress responses [38,39]. The presence ensures an increased osmotic adjustment response, heightened elimination of reactive oxygen species, and increased maintenance of the cellular energy state [40]. Some authors, mentions that an increase in photosynthetic activity increases sucrose; however, sucrose at high concentrations generates internal osmotic stress which can cause an imbalance in metabolite synthesis, and consequently, a reduction in biomass [41]. On the other hand, during the process of glycolysis, glucose is converted into pyruvic acid [42], which is a crucial component for the initiation of the Krebs cycle, this cycle is a major consumer of pyruvic acid and its supply must be coordinated with the demand of related products, so an imbalance in the synthesis and concentration of metabolites can cause a secondary effect on plant development, which would limit the final accumulation of biomass [43]. The above could be related to the biomass changes described above

On the other hand, the addition of CuCh, WH, and NE + CuC resulted in increased levels of the majority of the amino acids. In general, amino acid metabolism plays a significant role in plants’ energy metabolism. The energy status of plants is a fundamental factor affecting various physiological processes, including development and nutrient synthesis [44]. For example, the oxidation of tyrosine produces 34 adenosine triphosphate (ATP) molecules, and glutamine can be converted into γ-aminobutyric acid and subsequently transformed into succinic acid, which plays a role in the tricarboxylic acid (TCA) cycle [38]. Proline, on the other hand, plays an important role in redox buffering, energy transfer, and resistance against pathogens in plants [38,45].

Amino acids—including tyrosine, lysine, phenylalanine, and tryptophan—are biosynthetic precursors for alkaloids associated with plants’ defence mechanisms [46]. In light of this information and our findings, one can assume that higher concentrations of amino acids, such as phenylalanine, stimulate the synthesis of compounds derived from phenylpropanoids, such as flavonoids. These compounds play a crucial role in promoting robust crop development, particularly in challenging environmental conditions. Some authors suggested that stress conditions can enhance the accumulation of metabolites such as sugars and amino acids [47]. In this sense, NFs act as elicitors or stress-inducing agents that trigger diverse metabolic responses in plants [35]. That situation in turn leads to the accumulation or depletion of both general and specialised metabolites [20].

We also observed a significant increase in the concentration of nucleosides such as adenosine, guanine, uridine, and cytidine, as well as compounds like NAD^+^ and trigonelline, with the addition of CuCh. Nucleosides are essential components of nucleic acids, and they act as precursors of primary and specialised metabolites [20]. They also serve as integral components of enzyme cofactors and second messengers [48]. However, the accumulation of nucleosides generally exerts a negative impact on plants, as observed in the case of cytidine [49]. For example, NAD^+^ actively participates in catabolic processes, facilitating the generation of cellular energy, and it plays an important role in signalling pathways [50]. Trigonelline, on the other hand, acts as an osmolyte-enabling plant adaptation to environmental stressors [51]. Chitosan has a diverse chemical structure with variable compositions and molecular weight distributions, which contributes to its propensity to react easily with other active compounds [52,53]. The aforementioned characteristics may explain the observed increase in these variables that resulted the combination of Cu NPs and chitosan.

Nanofertilizers, in combination with biofertilizers, offer numerous advantages and open new perspectives for sustainable agriculture [54]. The application of metal nanoparticles together with biofertilizers can lead to the binding of plant proteins and metal nanoparticles. The proteins can act as encapsulating agents and form a layer on the surface of the metal nanoparticles. This protective layer prevents agglomeration, protects the nanoparticles from agglomeration and stabilizes the medium, which generates new actions and conditions in plants [55,56]. These effects can be favorable or unfavorable, or both. The above could explain the results found in our study as the combination of CuC and NE resulted in the highest concentration of metabolites in leaves, revealing a synergistic interaction. In contrast, an antagonistic effect was observed when CuCh was combined with WH.

The use of Cu at optimal concentrations as a NF promotes the growth and metabolism of plant such as onions, tomatoes, and alfalfa. This situation in turn leads to the induction of an antioxidant system and enhanced stress tolerance [57,58]. Furthermore, the accumulation of metabolites such as amino acids, organic acids, and sugars is contingent upon factors such as the developmental process, stage, leaf position on the stem, and harvest timing [41]. In addition, the increased levels of metabolites such as tryptophan, pyruvic acid, and sucrose promote seed formation and embryo growth through cell division, driven by metabolic interactions involved in the synthesis of plant hormones like auxins (indole-3-acetic acid via the indole-3-pyruvic acid pathway) and cytokinins, such as zeatin, which is synthesized from nucleosides [37,59]. In legume seeds, early embryo growth depends on the number of cells in the cotyledons, making the regulation of cell division critical during the initial stages of seed development [60]. This analysis enhances our understanding of the metabolic profile of black bean leaves and provides a valuable reference for improving regulation and producing higher-quality grains.

## 4. Materials and Methods

### 4.1. Experiment Development

We analysed “Michigan” variety black bean seeds. The crop was grown in the municipality of Tetepango, Hidalgo, Mexico (20°05’38.8” N, 99°09’57.4” W). The climate in Tetepango is dry, and the summer is rainy; the mean annual temperature and precipitation are 24 °C and 250 mm, respectively. The black beans were sown under rainfed conditions by directly planting them in the soil at the beginning of September 2022. The soil in Tetepango has a clay texture, an organic matter content of 3.29%, a neutral pH (7.2), an EC of 1.281 dS m^−1^, CEC of 38.02, and a high content of N-NO_3_^−^ (45.20 mg Kg^−1^), K^+^ (840.00 mg Kg^−1^), Ca^++^ (5900.00 mg Kg^−1^), Mg^+^ (730.00 mg Kg^−1^), and B (1.30 mg Kg^−1^). It has a low content of Na^+^ (97.00 mg Kg^−1^) and micro elements such as Fe^++^, Zn^++^, Mn^++^, and Cu^++^; it has 16.01 mg Kg^−1^ of usable P.

We evaluated the NFs and BFs separately, as well as in combination, analyzing a total of nine distinct treatments: copper nanoparticles synthesized with cotton (CuC), copper nanoparticles synthesized with chitosan (CuCh), nopal extract (NE), commercial Biojal^®^ worm humus (WH), and their respective combinations: NE + CuC, NuCh, WH + CuC, and WH + CuCh, along with an absolute control (C). The treatment concentrations were 50 mg L^−^¹ for CuC, CuCh, and NE, and 100 mL L^−^¹ for WH (Table S1).

The size of the Cu NPs, estimated using the X-ray powder diffraction technique (XRD), varied from 15 to 35 nm [61].

The NE contained N (600–670 mg L^−1^), P (154–170 mg L^−1^), Ca (1830–1950 mg L^−1^), Mg (700–800 mg L^−1^), Fe (5–10 mg L^−1^), Zn (>1 mg L^−1^), Cu (>1 mg L^−1^), Na (600 mg L^−1^), organic matter (250–302 mg L^−1^), humic acids (600 mg L^−1^), and total chlorophyll (4.7 mg L^−1^).

The WH contained NO_3_ (531.42 mg L^−1^), NH_4_ (212 mg L^−1^), phosphates (458.20 mg L^−1^), sulphates (1200.00 mg L^−1^), Ca (560.00 mg L^−1^), S (400.00 mg L^−1^), Cl^−^ (1701.60 mg L^−1^), Fe (53.00 mg L^−1^), Zn (1.00 mg L^−1^), Mg (100.00 mg L^−1^), Mn (1.00 mg L^−1^), B (12.00 mg L^−1^), and 0.54% organic matter.

Forty days after sowing (das), when the plants had two pairs of true leaves, the leaf application was carried out using a manual sprayer. We added 1% glacial acetic acid as a solubiliser to the NPs synthesised using chitosan. A total of two applications were carried separated by 15 days (i.e., 40 and 55 das).

The experimental design was a randomized complete block. Each block consisted of four 10 m furrows separated by 80 cm. There were three blocks in total. To evaluate the agronomic variables, 12 replicates per treatment were considered. Each replicate consisted of a single plant.

### 4.2. Agronomic Variables

Plant height (PH, cm) was determined using a flexometer, and stem diameter (SD, mm) was determined using a digital vernier caliper; the number of leaves per plant (NL) was determined by counting. To obtain the fresh weight (FW, g), the stem, leaves, and roots of each plant were weighed using a digital scale (Truper 102316 BASE-05B, Jilotepec, Mexico City, Mexico); the dry weight (DW, g) was obtained by drying the samples at room temperature.

### 4.3. NMR Analysis

For metabolomic analysis, 10 aqueous extracts per treatment were evaluated. Each extract was obtained from a single replicate. The sampling was carried out at 70 das. We targeted young leaves that were fully expanded. The collected samples were stored at −20 °C for 10 days and then lyophilised for 72 h at a temperature of −40 °C in a lyophiliser FreeZone 6 L-50C Console Freeze Dryer (Labconco, Kansas City, MO, USA); they were subsequently analysed using NMR. According to the methodology described [17], 50 mg of lyophilised tissue was weighed in 2 mL Eppendorf tubes, and 720 µL of deuterated water and 80 µL of potassium phosphate-buffer solution at a concentration of 0.7 mM and pH = 6 (7 mM TSP, 10 mM EDTA, 2 mM NaN3) were added. The resulting mixture was sonicated for 20 min (Branson 5510 Ultrasonic Cleaner, Marshall Scientific, Hampton, NH, USA) and centrifuged at 10,000 rpm (Prism^TM^ Microcentrifuge C2500, Labnet International Inc. Edison, NJ, USA) for 20 min at room temperature. Next, 600 μL of the supernatant was taken and transferred to a 5 mm NMR tube.

### 4.4. Nuclear Magnetic Resonance Analysis

The samples were analysed on a Bruker Ascend^TM^ 750 MHz spectrometer (Bruker Biospin, Rheinstetten, Germany) equipped with a TXI probe. The ^1^H NMR analyses were measured using the NOESYPR1D pulse sequence at 25 °C + 0.1 °K without rotation. The acquisition parameters were as follows: FID size = 64 K, number of dummy scans = 4, number of scans = 256, spectral width = 15.0 ppm, receiver gain = 32, acquisition time = 2.18 s, relaxation delay = 5.00 s, and FID resolution = 0.45 Hz.

### 4.5. Signal Assignments in NMR Spectra

Complete interpretation of ^1^H NMR spectra of the aqueous extract of leaves of black beans was achieved using 1D and 2D analyses such as ^13^C (Figure S3), COSY (Figure S4), HSQC (Figure S5), and HMBC (Figure S6). The spectral data of the metabolites identified in the leaves of black beans were compared with databases such as the Human Metabolome Data Base version 5.0 [62] and the Biological Magnetic Resonance Data Bank [63].

### 4.6. Spectral Processing and Quantification of Metabolites

We processed the NMR data according to the procedure [17]. Phase and baseline corrections were performed on the ^1^H NMR spectra using Mestrenova software (v.6.1.0 Mestrelab Research SL, Santiago de Compostela, Spain). The TSP signal was used to reference the spectra at 0.00 ppm and was normalised using the TSP signal intensity. The spectral intensities were reduced to integrated regions, called cubes, of equal width (0.04 ppm) for all spectral regions (0.50–10.00 ppm). The spectral region corresponding to the residual water signal (4.70–4.90 ppm) was excluded from the analysis. The resulting data matrix was subsequently used for the multivariate analysis [64].

Relative quantitation of metabolites was performed using the following expression [65]:

$$M_{x}=M_{y}\cdot \frac{I_{X}}{I_{Y}}\cdot \frac{N_{Y}}{N_{X}}$$

where *M_x_* = Relative concentration of metabolite; *M_y_* = Relative concentration of internal standard (TMSPd4); I_X_ = Relative integral value of the ^1^H NMR signal of metabolite; I_Y_ = Relative integral value of the ^1^H NMR signal of internal standard (TMSPd4); N_X_ = Number of protons belonging to the ^1^H NMR signal of metabolite; N_Y_ = Number of protons belonging to the ^1^H NMR signal of internal standard (TMSPd4).

### 4.7. Statistical Analysis

The statistical analysis was conducted using Infostat software (v 2020, Cordoba, Spain). Analysis of variance (ANOVA) was performed, followed by a Duncan test (*p* ≤ 0.05) for mean comparisons. The multivariate analysis was performed using SIMCA^®^-P v software. 14.1 (Umetrics, Umeå, Sweden). The data were normalized with a logarithmic function and subsequently scaled using Pareto scaling. Principal component analysis (PCA) was applied to the data set to identify differences or similarities between the treatments and explore intrinsic variations. The data sets were then subjected to orthogonal partial least squares discriminant analysis (OPLS-DA) to identify differential components among the samples and to determine the optimal relationship between independent and dependent variables. Model validation was carried out using permutation testing (200 times); the explained variables and the predictability of the models were verified using R^2^ and Q^2^ measures. A heat map was also created using MetaboAnalyst 3.0 software [66] to visualise the relative concentrations of metabolites resulting from each of the different treatments.

## 5. Conclusions

Nanofertilizers and biofertilizers are promising alternatives to conventional fertilizers given their ability to potentially mitigate the latter’s adverse impacts on agriculture and the environment. Both BFs and NFs are environmentally friendly and less detrimental than chemical fertilizers and pesticides; they are capable of boosting and stimulating optimal crop growth. However, when used in combination, they can exhibit either synergistic or antagonistic effects. For instance, the combination of CuC and NE in our study resulted in the highest concentration of metabolites in the leaves; that finding revealed a synergistic interaction. Conversely, an antagonistic effect was observed when CuCh was combined with WH. The results of this research may contribute to the identification and selection of suitable NFs and BFs and optimal combinations thereof to improve crop development and ensure higher seed yield and quality. However, due to the unique physicochemical properties of nanomaterials, they often have toxic consequences, the size and large surface area of these materials facilitate their dispersion and invasion into the human body, resulting in nanotoxicity, so precautionary measures should be taken before implementing nanoparticles on a large scale for agricultural uses, as these agricultural products will be consumed by humans and animals. Finally, the study of plant metabolites is crucial for understanding plant biology and unlocking their potential for various agronomic applications.

## Data Availability

The original contributions presented in this study are included in the article/Supplementary Materials. Further inquiries can be directed to the corresponding authors.

## Supplementary Materials

The following supporting information can be downloaded at: https://www.mdpi.com/article/10.3390/ijms26104844/s1.

## Abbreviations

The following abbreviations are used in this manuscript:

NFs	nanofertilizers
NPs	nanoparticles
BFs	biofertilizers
CuC	copper nanoparticles synthesised using cotton
CuCh	copper nanoparticles synthesised using chitosan
NE n	Nopal extract
WH c	commercial Biojal® worm humus
NAD^+^	nicotinamide adenine dinucleotide
Cu	copper
CuO	copper oxide
NMR	nuclear magnetic resonance
PH	plant height
SD	stem diameter
NL	number of leaves per plant
DW	dry weight
FW	fresh weight
PCA	principal component analysis
OPLS-DA	orthogonal partial least squares discriminant analysis
PC1	first principal component
PC2	second principal component
IAA	indole-3-acetic acid
ATP	adenosine triphosphate
TCA	tricarboxylic acid
