# Supplementary material for: NMR-Based Metabolomic Approach to Study Growth of Phaseolus vulgaris L. Seedlings Through Leaf Application of Nanofertilizers and Biofertilizers
Source: Int J Mol Sci. 2025 May 19;26(10):4844. doi: 10.3390/ijms26104844 (PMC12112589; doi:10.3390/ijms26104844)
Supplement: Supplementary file 1 [file ijms-26-04844-s001.zip › ijms-3520875-supplementary.pdf]

SUPPLEMENTARY INFORMATION

A NMR-based Metabolomic Approach to Study Growth of Phaseolus vulgaris L. Seedlings through Leaf Application of Nanofertilizers and Biofertilizers

* Corresponding autor
Alma Delia Hernández-Fuentes, almah@uaeh.edu.mx
Elvia Becerra-Martínez, elmartinezb@ipn.mx

INDEX

Table S1. Nanofertilizers, biofertilizers and their respective combinations for foliar application on black bean seedlings (*Phaseolus vulgaris* L.).

Table S2. ^{1}H NMR chemical shifts, assignment, and multiplicity (D2O) of the metabolites Identified in aqueous extracts of black bean (*Phaseolus vulgaris* L.) leaves treated with NFs and BFs.

Table S3. R^2 and Q^2 values with their corresponding CV-ANOVA, PCA, and OPLS-DA permutations obtained from black bean (*Phaseolus vulgaris* L.) leaf accession models.

Fig. S1. Effect of NFs and BFs on agronomic variables in black bean seedlings (*Phaseolus vulgaris* L.).

Fig. S2. ^{1}H NMR spectrum of the aqueous extracts of black bean (*Phaseolus vulgaris* L.) leaves without treatment. The spectrum obtained at 750 MHz was extended from 0.00 to 9.50 ppm (A); from 0.80 to 3.30 ppm (B); from 3.30 to 5.50 ppm (C) and from 5.50 to 9.50 ppm (D). Signal assignments are based on 2D NMR experiments and the literature (Hernández-Guerrero et al., 2021).

Fig. S3. ^{13}C NMR spectrum obtained at 188.62 MHz from aqueous extracts of black bean (*Phaseolus vulgaris* L.) leaves without treatment.

Fig S4. ^{1}H-^{1}H COSY *spectrum* of the aqueous extracts of black bean (*Phaseolus vulgaris* L.) leaves without treatment.

Fig. S5. ^{1}H-^{13}C HSQC *spectrum* of the aqueous extracts of black bean (*Phaseolus vulgaris* L.). **Fig S6.** ^{1}H-^{13}C HMBC *spectrum* of the aqueous extracts of black bean (*Phaseolus vulgaris* L.) leaves without treatment.

Fig S6. 1H-13C HMBC spectrum of the aqueous extracts of black bean (Phaseolus vulgaris L.) leaves without treatment.

Fig. S7. Representative ^{1}H NMR spectra of the aqueous extracts of black bean (*Phaseolus vulgaris* L.) leaves treated with NFs and BFs. C: absolute control; NPsCuC: copper-cotton NPs; NPsCuCh: copper-chitosan NPs; NE: nopal extract; WH: Biojal® worm humus.

Table S1. Nanofertilizers, biofertilizers and their respective combinations for foliar application on black bean seedlings (*Phaseolus vulgaris* L.).

<table>
<tr><th></th><th>Treatments</th><th>Nanofertilizers (Dose)</th><th>Biofertilizers (Dose)</th></tr>
<tr><td>T1</td><td>CuC</td><td rowspan="4">CuC (50 mg L^{-1})</td><td rowspan="4">NE (50 ml L^{-1})</td></tr>
<tr><td>T1</td><td>CuCh</td></tr>
<tr><td>T3</td><td>NE</td></tr>
<tr><td>T4</td><td>WH</td></tr>
<tr><td>T5</td><td>NE+CuC</td><td rowspan="4">CuCh (50 mg L^{-1})</td><td rowspan="4">WH (100 ml L^{-1})</td></tr>
<tr><td>T6</td><td>WH+CuC</td></tr>
<tr><td>T9</td><td>NE+CuCh</td></tr>
<tr><td>T8</td><td>WH+CuCh</td></tr>
<tr><td>T9</td><td>C</td><td colspan="2">Distilled water</td></tr>
</table>

C: Absolute control
CuC: copper-cotton nanoparticles;
CuCh: copper-chitosan nanoparticles;
NE: nopal extract;
WH: commercial worm-humus Biojal®

Table S2. ^{1}H NMR chemical shifts, assignment, and multiplicity (D_2O) of the metabolites identified in aqueous extracts of black bean (*Phaseolus vulgaris* L.) leaves without treatment.

	Metabolite	Chemical shifts (ppm), J (Hz), multiplicity
		**Sugars**
1	Arabinose	5.25 (d, *J* = 3.5)
2	Fructose	3.98 (m), 4.01 (dd, *J* = 12.7, 1.3 Hz)
3	Galactose	4.57 (d, *J* = 7.9 Hz)
4	Glucose	4.63 (d, *J* = 7.9 Hz), 5.22 (d, *J* = 3.7 Hz).
5	Mannose	5.17 (d, *J* = 1.23 Hz)
6	Myo-inositol	3.26 (t, *J* = 9.4 Hz)
7	Sucrose	4.20 (d, *J* = 8.8 Hz), 5.40 (d, *J* = 3.8 Hz) CH-7
		**Amino acids**
8	Alanine	1.47 (d, *J* = 7.2 Hz)
9	Arginine	1.72 (m), 1.91 (m), 3.20 (t, *J* = 6.9)
10	Asparagine	2.85 (dd, *J* = 16.9, 7.8 Hz), 2.94 (dd, *J* = 16.9, 4.2 Hz)
11	Aspartic acid	2.68 (dd, *J* = 17.5, 7.4 Hz), 2.80 (dd, *J* = 17.5, 3.7 Hz)
12	GABA	1.89 (m), 2.29 (t, *J* = 7.4 Hz)
13	Glutamic acid	2.15 (m), 2.36 (m)
14	Glutamine	2.14 (m), 2.46 (m)
15	Histidine	8.30 (m), 7.25 (m)

16	Isoleucine	0.92 (t, *J* = 7.4 Hz), 1.00 (d, *J* = 7.0 Hz)
17	Leucine	0.94 (d, *J* = 6.2 Hz) , 0.95 (d, *J* = 6.2 Hz)
18	Methionine	2.12 (s), 2.65 (t, *J* = 7.6 Hz)
19	Phenylalanine	7.31 (d, J = 7.5), 7.36 (m), 7.41 (t, *J* = 7.5 Hz)
20	Proline	2.01 (m), 2.3 (m)
21	Threonine	1.32 (d, *J* = 6.6 Hz)
22	Tryptophan	7.52 (d, *J* = 8.0 Hz), 7.71 (d, *J* = 8.0 Hz)
23	Tyrosine	7.18 (d, *J* = 7.18 Hz), 6.87 (d, *J* = 7.18 Hz)
24	Valine	0.98 (d, *J* = 7.0 Hz), 1.03 (d, *J* = 7,0 Hz)
		**Organic acids**
25	Acetic acid	1.92 (s)
26	Citric acid	2.52 (d, *J* = 15.6 Hz), 2.71 (d, *J* = 15.6 Hz)
27	Formic acid	8.44 (s)
28	Fumaric acid	6.51 (s)
29	Malic acid	2.53 (dd, *J* = 15.5, 9.9 Hz), 2.69 (dd, *J* = 15.5, 3.2 Hz)
30	Pyruvic acid	2.34 (s)
31	Succinic acid	2.43 (s)
32	Tartaric acid	4.38 (s)
33	2-Hydroxyisobutyric acid	1.36 (s)
34	3-O-Caffeoylquinic acid	6.42 (d, *J* = 15.9 Hz)
35	4-O-Caffeoylquinic acid	6.46 (d, *J* = 15.9 Hz)

36	5-O-Caffeoylquinic acid	6.45 (d, J = 15.9 Hz)
	**Alcohols**	
37	Methanol	3.34 (s)
	**Nucleosides**	
38	Adenosine	6.06 (d, J = 6.1 Hz), 8.17 (s), 8.22 (s)
39	Cytidine	6.05 (d, J = 7.6 Hz), 7.84 (d, J = 7.6 Hz)
40	Guanosine	5.90 (d, J = 6.0 Hz), 7.78 (s)
41	Uridine	5.88 (m), 5.90 (m), 7.86 (d, J = 8.1 Hz)
	**Other compounds**	
42	Choline	3.18 (s)
43	NAD^+	9.45 (s)
44	Trigonelline	8.83 (m), 9.11 (s)

Table S3. R^2 and Q^2 values with their corresponding CV-ANOVA, PCA, and OPLS-DA permutations obtained from black bean (*Phaseolus vulgaris* L.) leaf accession models.

	PCA		OPLS-DA			CV-ANOVA	Permutation
	R^2X	Q^2	R^2X	R^2Y	Q^2		
**All groups**	0.777	0.524	0.717	0.291	0.226	6.30E-06	✓
**T vs CuA vs CuQ vs N vs L**	0.839	0.528	0.776	0.682	0.599	6.39E-12	✓
**TvsN+CuAvsL+CuAvsN+CuQvsL+CuQ**	0.823	0.589	0.806	0.791	0.665	1.82E-24	✓

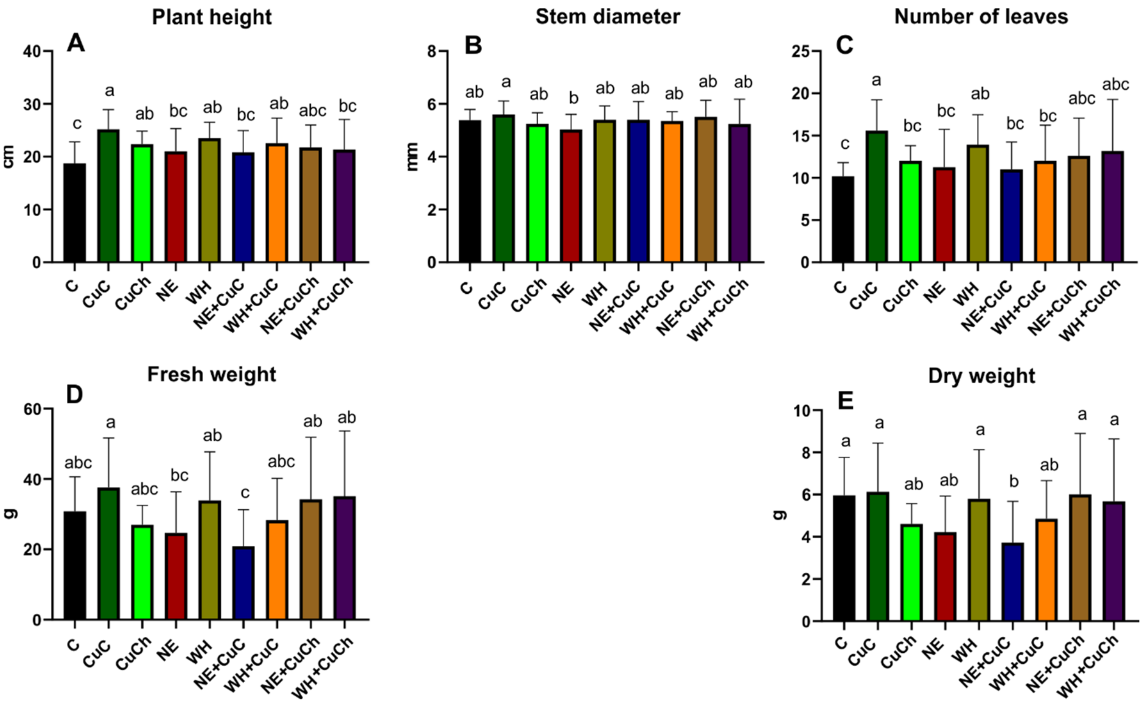

Fig. S1. Effect of NFs and BFs on agronomic variables in black bean seedlings (*Phaseolus vulgaris* L.).

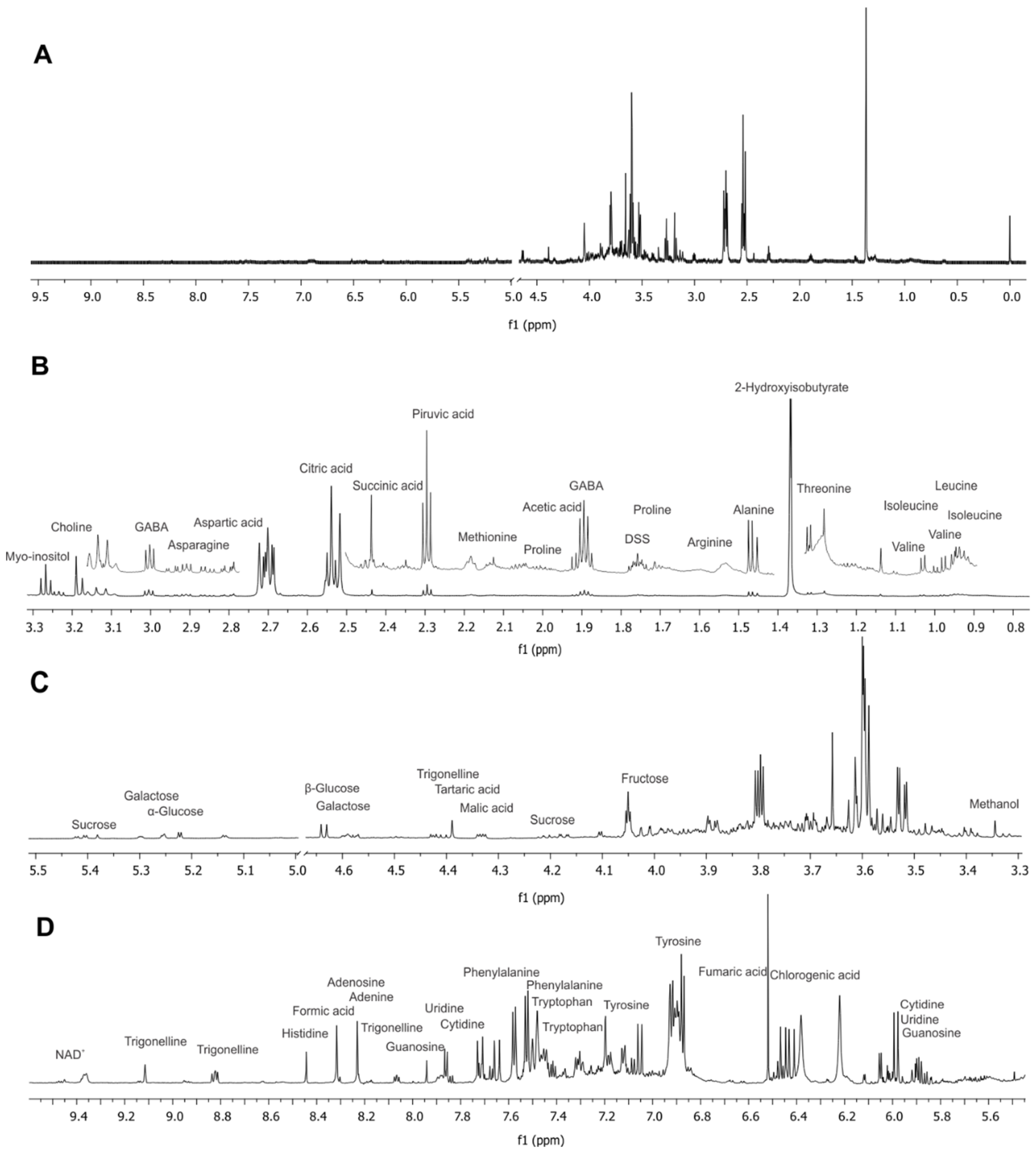

Fig. S2. ^{1}H NMR spectrum of the aqueous extracts of black bean (*Phaseolus vulgaris* L.) leaves without treatment (C). The spectrum obtained at 750 MHz was extended from 0.00 to 9.50 ppm (A); from 0.80 to 3.30 ppm (B); from 3.30 to 5.50 ppm (C) and from 5.50 to 9.50 ppm (D). Signal assignments are based on 2D NMR experiments and the literature (Hernández-Guerrero et al., 2021).

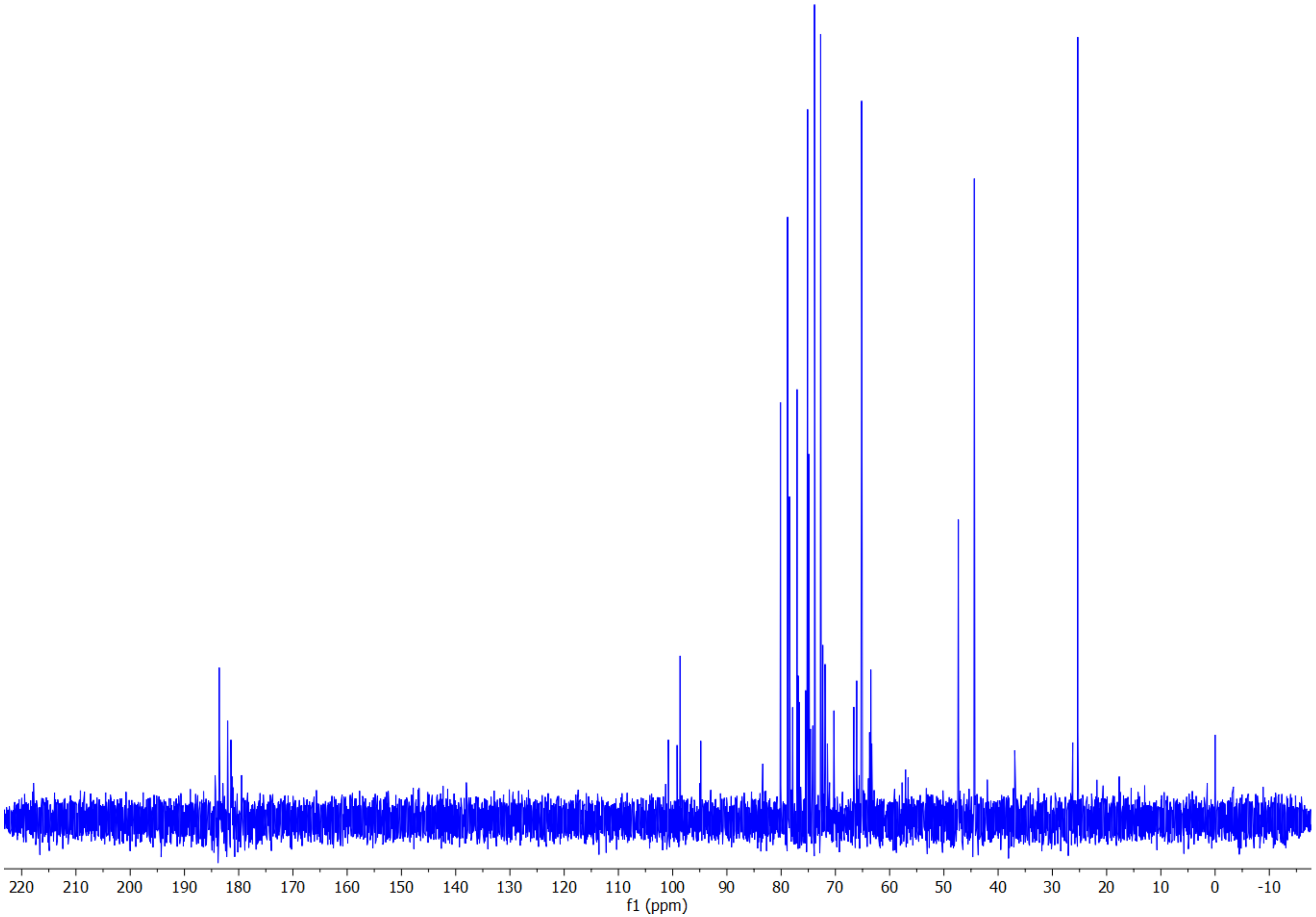

Fig. S3. ^{13}C NMR spectrum obtained at 188.62 MHz from aqueous extracts of black bean (*Phaseolus vulgaris* L.) leaves without treatment (C).

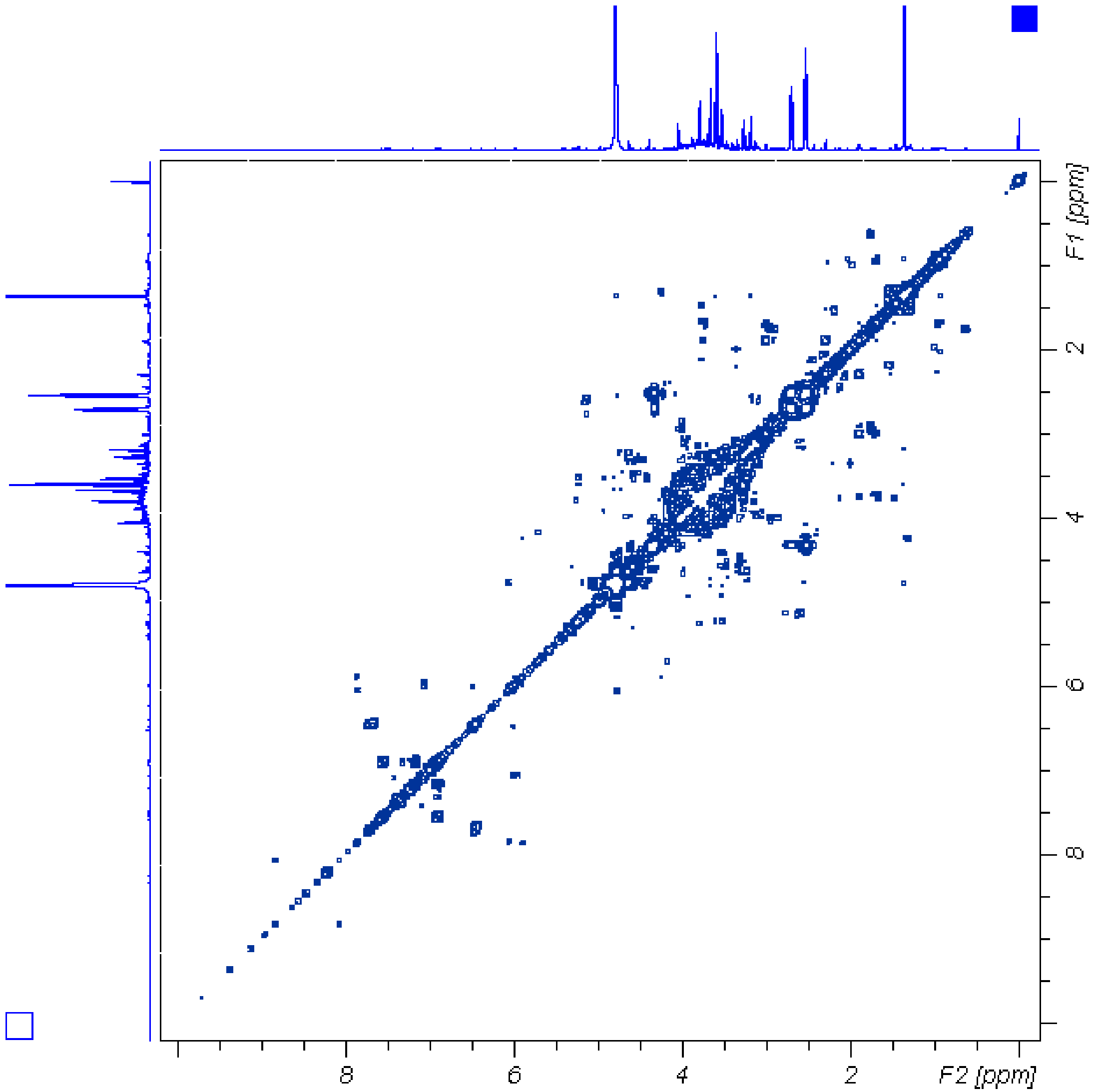

Fig S4. ^{1}H-^{1}H COSY *spectrum* of the aqueous extracts of black bean (*Phaseolus vulgaris* L.) leaves without treatment (C).

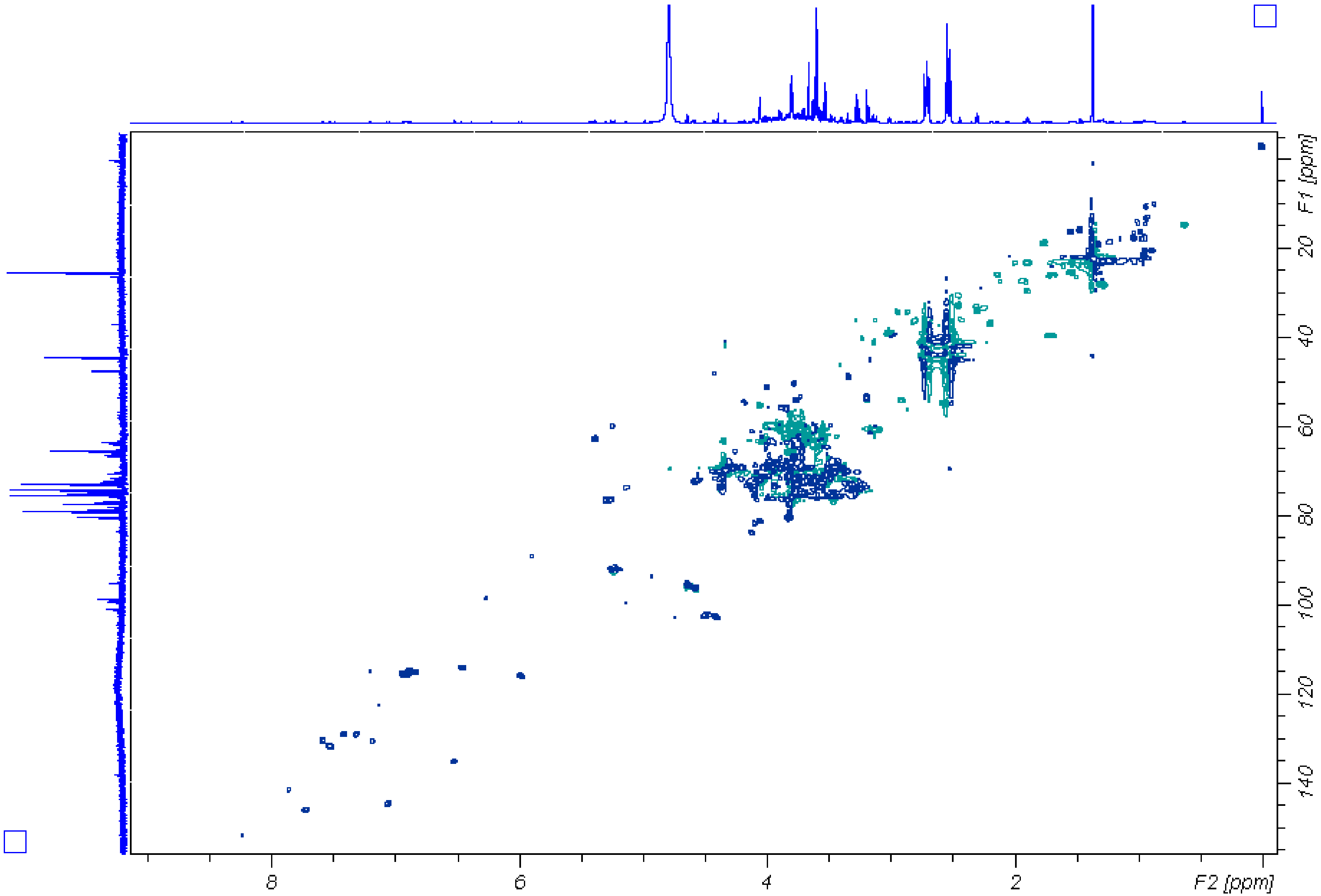

Fig. S5. ^{1}H-^{13}C HSQC *spectrum* of the aqueous extracts of black bean (*Phaseolus vulgaris* L.) leaves without treatment (C).

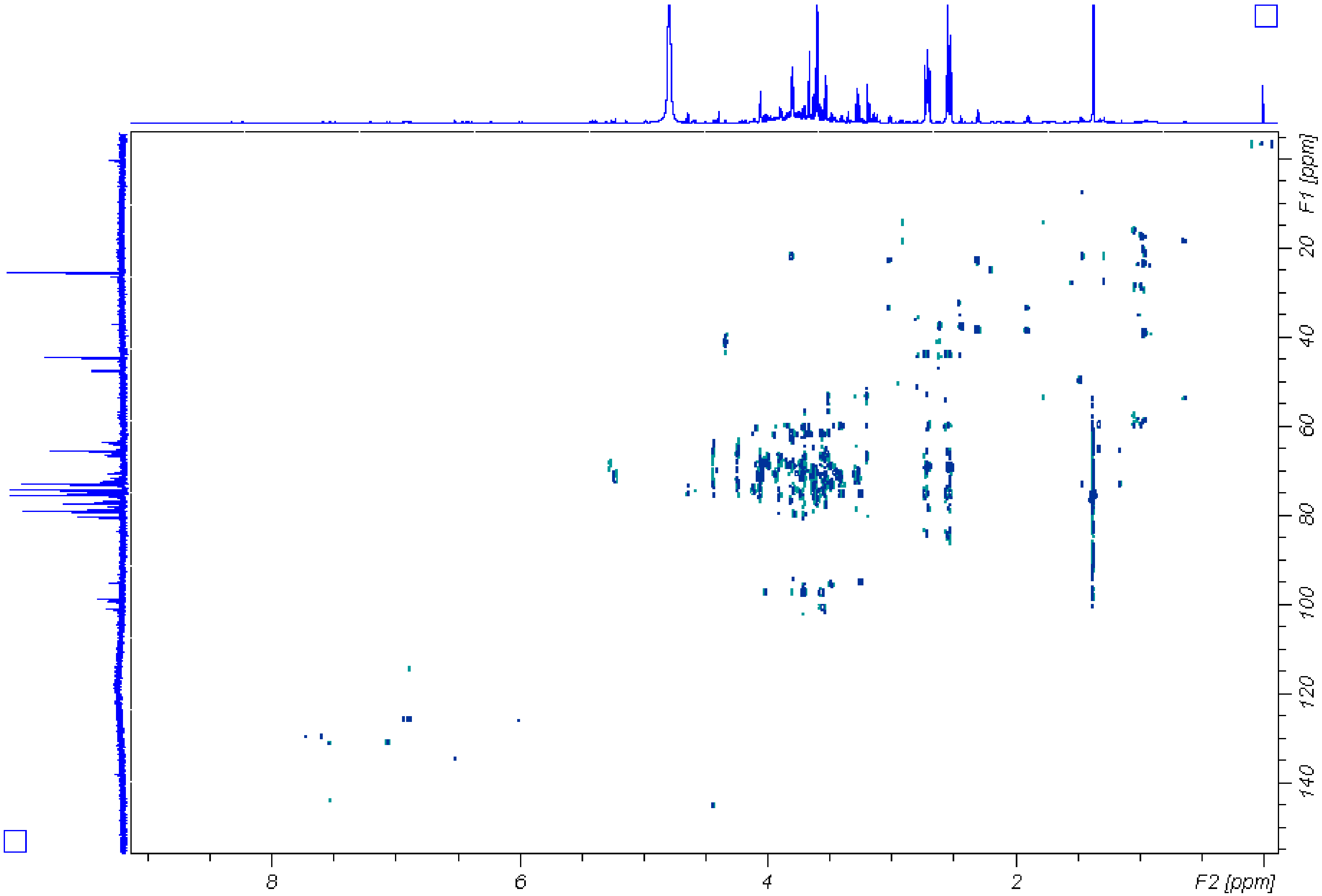

Fig S6. ^{1}H-^{13}C HMBC *spectrum* of the aqueous extracts of black bean (*Phaseolus vulgaris* L.) leaves without treatment (C).

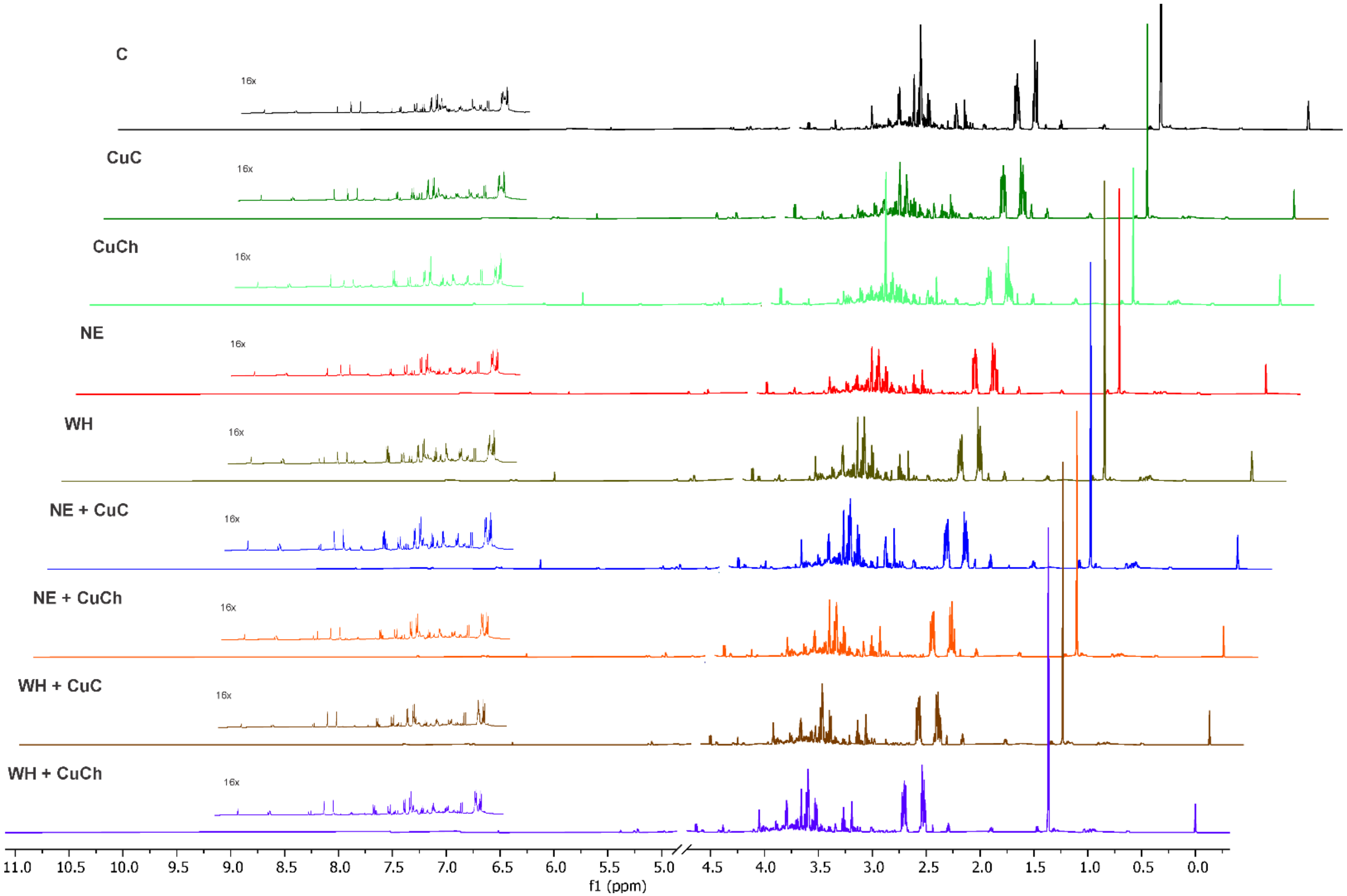

Fig. S7. Representative ^{1}H NMR spectra of the aqueous extracts of black bean (*Phaseolus vulgaris* L.) leaves treated with NFs and BFs. C: absolute control; CuC: copper-cotton NPs; CuCh: copper-chitosan NPs; NE: nopal extract; WH: Biojal® worm humus.
